# Characterizing the original anti‐C5 function‐blocking antibody, BB5.1, for species specificity, mode of action and interactions with C5

**DOI:** 10.1111/imm.13228

**Published:** 2020-07-13

**Authors:** Wioleta M. Zelek, Georgina E. Menzies, Andrea Brancale, Brigitta Stockinger, Bryan Paul Morgan

**Affiliations:** ^1^ Systems Immunity University Research Institute School of Medicine Cardiff University Cardiff UK; ^2^ Dementia Research Institute Cardiff University Cardiff UK; ^3^ School of Pharmacy and Pharmaceutical Sciences Cardiff University Cardiff UK; ^4^ The Francis Crick Institute London UK

**Keywords:** complement, complement therapeutics, eculizumab, monoclonal antibody, mouse C5, mouse models

## Abstract

The implication of complement in multiple diseases over the last 20 years has fuelled interest in developing anti‐complement drugs. To date, the focus has been on C5; blocking cleavage of C5 prevents formation of two pro‐inflammatory activities, C5a anaphylatoxin and membrane attack complex. The concept of C5 blockade to inhibit inflammation dates back 30 years to the description of BB5.1, an anti‐C5 blocking monoclonal antibody raised in C5‐deficient mice. This antibody proved an invaluable tool to demonstrate complement involvement in mouse disease models and catalysed enthusiasm for anti‐complement drug development, culminating in the anti‐human C5 monoclonal antibody eculizumab, the most successful anti‐complement drug to date, already in clinical use for several rare diseases. Despite its key role in providing proof‐of‐concept for C5 blockade, the mechanism of BB5.1 inhibition remains poorly understood. Here, we characterized BB5.1 cross‐species inhibition, C5 binding affinity and chain specificity. BB5.1 efficiently inhibited C5 in mouse serum but not in human or other rodent sera; it prevented C5 cleavage and C5a generation. BB5.1 bound the C5 *α*‐chain with high affinity and slow off‐rate. BB5.1 complementarity‐determining regions were obtained and docking algorithms were used to predict the likely binding interface on mouse C5.

## Graphical abstract

The work provides a mechanistic basis for the activities of this landmark antibody BB5.1, a much‐used tool that was a major impetus to the development of anti‐complement drugs for human disease.

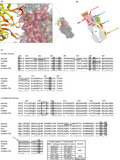

**Abbreviations**
Abs: absorbance
AP: alternative pathway
BSA: bovine serum albumin
C5: complement component 5
CP: classical pathway
ELISA: enzyme‐linked immunosorbent assay
HBS: HEPES‐buffered saline
mAb: monoclonal antibody
MAC: membrane attack complex
PBS: phosphate‐buffered saline
RbE: rabbit erythrocytes
RT: room temperature
ShEA: antibody‐sensitized sheep erythrocytes
WB: Western blot

## Introduction

Complement is a key component of the immune system, evolved to protect from bacterial infections; however, dysregulation of complement drives inflammation and leads to pathology in many diseases.^1^, ^2^ Activation of complement by way of classical, lectin or alternative pathways triggers enzymatic cascade reactions that all result in formation of C3‐cleaving enzymes (convertases) and subsequently C5 convertases; these cleave C5 into C5a, a potent anaphylatoxin, and C5b, which nucleates formation of membrane attack complex (MAC) by sequentially binding C6 and C7. The C5b67 complex binds membranes and sequentially recruits C8 and C9 to complete the MAC.^2^, ^3^ Among the array of complement proteins, regulators and receptors, C5 plays a major role in complement‐mediated inflammation and for that reason has been the favoured target for the development of anti‐complement drugs. Since the anti‐C5 monoclonal antibody (mAb) eculizumab entered the clinic 12 years ago, the field has grown to the point where a recent compendium listed 28 anti‐complement drugs in development; of these, 12 target C5.^2^

The first disease targets for anti‐complement drugs were rare complement‐driven diseases caused by complement gene mutations or polymorphisms, notably paroxysmal nocturnal haemoglobinuria and atypical haemolytic uraemic syndrome,^4^, ^5^, ^6^, ^7^ but complement is also implicated in many more common diseases, including age‐related macular degeneration, myasthenia gravis, and in multiple central nervous system diseases including Alzheimer's disease, neuromyelitis optica and multiple sclerosis.^1^, ^8^ In haemolytic uraemic syndrome and paroxysmal nocturnal haemoglobinuria, blocking MAC assembly with the anti‐C5 mAb eculizumab prevents pathology and transforms patient outcomes.^4^, ^5^, ^6^, ^7^

The evidence underpinning the rapid developments in complement therapeutics has come from animal studies; a large proportion of these studies have used the same key agent, a function‐blocking anti‐C5 mAb BB5.1. First reported over 30 years ago, BB5.1 was generated by immunization of C5‐deficient mice and blocked haemolysis in normal mouse serum.^9^ BB5.1 not only provided a strong proof‐of‐concept for the therapeutic impact of inhibition of C5a/MAC, but also focused attention on C5 as target. BB5.1 was tested in mouse models of arthritis, renal injury, myasthenia gravis, multiple sclerosis, transplantation, immune complex disease, ischaemia–reperfusion injury, uveitis, colitis, meningitis, sepsis and pemphigus; in this long list of diverse model diseases BB5.1 was effective and safe.^10^, ^11^, ^12^, ^13^, ^14^, ^15^, ^16^, ^17^, ^18^, ^19^

The success of BB5.1 provided the rationale for the generation of human‐specific blocking anti‐C5 mAb. Indeed, as early as 1996, a human C5 blocking mAb 5G1.1 was described that was modified, humanized and finally marketed as eculizumab in 2002.^20^, ^21^, ^22^, ^23^ Eculizumab binds the C5*α*‐chain MG7 domain at an epitope spanning K879 to R885, remote from the *α*‐chain C5 convertase cleavage site (R751–L752), acting as a conformational lock and preventing C5 from binding the convertase.^4^, ^23^, ^24^, ^25^ Recent reports highlight the complexity of the eculizumab epitope on C5, with multiple residues outside the K879–R885 epitope playing important roles; for example, residues T916 and W917, which are unique to human C5, are involved in eculizumab binding and probably explain the lack of cross‐reactivity of this mAb with C5 from other species, including non‐human primates.^26^, ^27^

The historical importance of BB5.1 as a catalyst for the development of anti‐complement drugs is clear and it remains a useful and popular tool for proof‐of‐concept studies in different models; however, its binding characteristics and mechanism of inhibition remain unknown. Here, we characterized the BB5.1 mAb, including its binding to and inhibition of C5 from different species, its binding affinities for mouse C5, its mechanism of action and its C5‐binding interface. We show that Fab fragments of the mAb are efficient C5 inhibitors and provide complementarity‐determining region (CDR) sequence data that enabled *in silico* prediction of its binding site on C5 and will facilitate future modifications of this unique mAb. We anticipate that a better understanding of the mechanism of action of this venerable mAb will further enhance its usefulness for the research community.

## Materials and methods

All chemicals, except where otherwise stated, were obtained from either Fisher Scientific UK (Loughborough, UK) or Sigma Aldrich (Gillingham, UK) and were of analytical grade. All tissue culture reagents and plastics were from Invitrogen Life Technologies (Paisley, UK). Sheep, rabbit and guinea pig erythrocytes in Alsever's solution were from TCS Biosciences (Claydon, UK). Eculizumab was donated by Professor David Kanavagh (Newcastle University, UK), and the novel anti‐C5 mAb crovalimab (RO7112689) was donated by Roche Diagnostics (Basel, Switzerland).

Human and animal (mouse, rat, rabbit and guinea pig) sera were prepared in‐house from freshly collected blood. For all species except mouse, blood was clotted at room temperature for 1 hr, and then placed on ice for 2 hr for clot retraction before centrifugation and harvesting of serum. For mouse, blood was placed on ice immediately after harvest and clotted for 2 hr on ice before serum harvest. Sera were stored in aliquots at −80° and not subjected to freeze–thaw cycles.

#### Production and isotyping of BB5.1

The hybridoma cell line producing BB5.1 was re‐cloned and expanded; the antibody (mAb) was produced in large quantities using Integra flasks [Integra Biosciences (Tathcham, Berkshire, UK), Generon, CeLLine 1000 DC‐90005) in medium supplemented with ultralow IgG fetal bovine serum (ThermoFisher, Loughborough, UK), and purified under sterile conditions on a 5‐ml HiTrap Protein G sepharose column (GE Healthcare, Amersham, UK; #GE17‐0405‐01). Purity of the mAb was confirmed by sodium dodecyl sulphate–polyacrylamide gel electrophoresis (SDS–PAGE) and the isotype was tested using IsoStrips (#11493027001; Roche).

#### Haemolytic assays

The inhibitory activity of BB5.1 in human and animal sera was investigated using haemolysis assays. For the classical pathway (CP; CH50) assay, sheep erythrocytes (ShE) were sensitized by incubation using rabbit anti‐ShE antiserum (#ORLC25, Siemens Amboceptor; Cruinn Diagnostics, Dublin, UK; ShEA), then suspended in HEPES‐buffered saline (HBS) containing Ca^2+^ and Mg^2+^ at 2% (vol:vol); for measurement of CP activity in male mouse serum, ShEA were additionally incubated with mouse anti‐rabbit IgG (#3123; Invitrogen; 25 µg/ml) for 30 min at 37° before washing and re‐suspending in HBS.^28^ Serum dilutions for each species were selected in preliminary experiments to give near complete haemolysis in the CP assay in the absence of test mAb: normal human serum, 2·5%; normal male mouse serum, 25% (using the double‐sensitized cells as described above); normal rat serum, 2·5%; normal guinea pig serum, 2·5%; normal rabbit serum, 25%. A serial dilution series of BB5.1 mAb (667–0 nm for intact mAb; 2000–0 nm for Fab) was prepared in HBS and aliquoted in triplicate into a 96‐well round‐bottomed plate at 50 µl/well, then serum at the appropriate dilution and 2% ShEA (50 µl/well of each; double‐sensitized for mouse as above) was added. Plates were incubated at 37° for 30 min, centrifuged and haemoglobin in the supernatant was measured by absorbance at 405 nm. For the alternative pathway (AP; AH50) haemolysis assay, unsensitized rabbit erythrocytes (RbE) were suspended in HBS containing 5 mm EGTA and 3 mm MgCl_2_ at 2% (vol:vol). Lytic serum dose was set and test mAbs were titrated for inhibition essentially as described for the CP assay. For each assay, percentage lysis was calculated according to: % Lysis = Absorbance (Abs) sample − Abs background)/(Abs max − Abs background) × 100%. graphpad prism (v. 5.0) was used for data analysis (GraphPad, San Diego, CA).

#### Characterization of BB5.1 by ELISA

Direct enzyme‐linked immunosorbent assay (ELISA) was used to test whether BB5.1, either intact mAb or Fab fragments, bound mouse or human C5, as previously described.^29^ Maxisorp (Nunc, Loughborough, UK) 96‐well plates were coated with mouse or human C5 (purified in‐house; 0·5 µg/ml in bicarbonate buffer, pH 9·6) at 4° overnight; wells were blocked [1 hr at 37° with 2% bovine serum albumin (BSA) in phosphate‐buffered saline (PBS)] and washed in PBS containing 0·05% Tween20 (PBS‐T). Dilutions of purified BB5.1, intact mAb or Fab; 5–0 and 20–0 µg/ml, respectively (stock concentrations of all proteins established using the BCA assay), in 0·2% BSA‐PBS, were added in triplicate to wells coated with mouse or human C5 and incubated for 1 hr at 37°. Wells were washed with PBS‐T then incubated (1 hr, 37°) with secondary antibody Peroxidase AffiniPure Donkey Anti‐Mouse IgG (H + L) (minimal cross‐reactivity: bovine, chicken, goat, guinea pig, Syrian hamster, horse, human, rabbit, sheep serum proteins) or Peroxidase AffiniPure F(ab')_2_ Fragment Donkey Anti‐Human IgG (H + L) (minimal cross‐reactivity: bovine, chicken, goat, guinea pig, Syrian hamster, horse, mouse, rabbit, rat, sheep serum proteins) horseradish peroxidase (HRP) labelled; 715‐035‐150; 709‐036‐149; Jackson ImmunoResearch, Ely, UK) for 1 hr at 37°. After washing, plates were developed using *O*‐phenylenediamine dihydrochloride (Sigma‐Aldrich) and absorbance (492 nm) was measured. graphpad prism (v. 5.0) was used for data analysis.

#### Generation of BB5.1 Fab fragments

Fab fragments were generated from intact mAb (Pierce, Amersham, UK; #44980) to test C5 binding capacity and inhibitory activity after the enzymatic digestion. BB5.1 was buffer exchanged using zebra spin desalting columns (7KMWCO; Pierce; #89889) to freshly prepared digestion buffer (1 mm EDTA, 50 mm sodium phosphate, 2 mm Cysteine‐HCl‐H_2_O, pH7). Immobilized ficin (1 ml of suspension; Pierce; #44881), recommended for IgG1 mAb, was washed by centrifugation in digestion buffer, re‐suspended in 2 ml of buffer‐exchanged mAb (10 mg), then incubated for 2 hr at 37° followed by 24 hr incubation at room temperature, with gentle mixing during the incubation. The resin was washed three times by centrifugation with 5 ml PBS, the wash fractions containing the digested antibody combined and applied to a Protein A or G column (GE Healthcare; # 17‐0402‐01, # 29‐0485‐81), to remove Fc‐containing proteins according to the manufacturer’s instruction. The run‐through containing Fab fragments was collected, and bound Fc and residual intact mAb were eluted using 0·1 m glycine pH 2·5. The Fab fragments were analysed on SDS–PAGE under non‐reducing and reducing conditions, dialysed against HBS buffer and stored at −20°.

#### Characterization of BB5.1 by Western blot

Mouse and human C5 (in‐house; 1 µg) were resolved on SDS–polyacrylamide gels under reducing (5% *β*‐mercaptoethanol) and non‐reducing conditions, then electrophoretically transferred onto a 0·45‐µm nitrocellulose membrane (GE Healthcare). After transfer, non‐specific sites on the membrane were blocked with 5% BSA in PBS‐T. After washing in PBS‐T, membranes were incubated for1 hr at room temperature with biotinylated BB5.1 (1 µg/ml; Pierce, #21327) in 5% BSA PBS‐T. After washing, bound biotinylated test mAb was detected with Streptavidin‐HRP (R&D Systems, Abingdon, UK; # 890803) 1 : 5000 dilution in 5% BSA PBS‐T, incubated for 40 min at RT. After washing, the blot was developed with enhanced chemiluminescence (GE Healthcare) and visualized by autoradiography.

#### Testing whether BB5.1 inhibits generation of C5a from mouse C5 by the CVF C3/C5 convertase

Mouse C5 was mixed with BB5.1 at 1 : 5 molar excess in HBS, or with OmCI (tick saliva C5‐blocker; *Ornithodoros moubata*; coversin) at 1 : 10 molar ratio, then incubated for 30 min at room temperature. Cobra venom factor (CVF from *Naja naja kaouthia*) C3/C5 convertase (CFVBb; Mr of complex ~ 205 000) was generated by mixing CVF with human factor B (FB) and factor D (FD) at a 1 : 1 : 0·1 molar ratio in HBS containing 5 mm MgCl_2_ and incubated for 1 hr at 37°.^30^ CVF and FB were in‐house purified as described previously;^31^ FD was purchased from CompTech (Tyler, TX; A136). Preassembled CVF convertases were added to the pre‐incubated BB5.1–C5 and OmCI–C5 complexes or C5 at 1 : 10 molar ratio and incubated at 37°; aliquots were taken at 0, 1 and 12 hr. For Western blot, samples were diluted 1 in 3 in HBS, separated and transferred as above, then probed with rabbit polyclonal goat anti‐human C5 at 2 µg/ml (CompTech; A220), detected using rabbit anti‐goat‐HRP conjugate at 1 : 10 000 dilution (# 305‐035‐045; Jackson ImmunoResearch). Positive controls included CVFBb incubated with mouse C5 for 12 hr, intact mouse C5 (in house), C5a (Comptech, A144) diluted in HBS.

#### Surface plasmon resonance measurement of BB5.1 binding affinity to mouse C5

Surface plasmon resonance (SPR) binding analyses were carried out on a Biacore T200 instrument (GE Healthcare). BB5.1 was immobilized directly onto the CM5 sensor chip by amine coupling (#29‐1496‐03; GE Healthcare) at approximately 200 RU. Mouse C5 in HBS (10 mm HEPES, pH 74, 150 mm NaCl, 0·05% surfactant P20; HBS‐EP) was flowed over the immobilized BB5.1 in a concentration series from 52 to 4 nm and interactions with the immobilized mAbs were analysed. For kinetic analysis, the flow rate was maintained at 30 µl/min, and data were collected at 25°. Data from a reference cell were subtracted to control for bulk refractive index changes. The *R*_max_ was kept low and the flow rate was kept high to eliminate mass transfer. All reagents used were of high purity and polished by size exclusion chromatography immediately before use to ensure removal of any aggregates. Data were evaluated using biacore evaluation software (GE Healthcare).

#### BB5.1 sequence determination and identification of CDR

Total mRNA was extracted from the BB5.1‐producing hybridoma and sent for antibody sequencing by whole transcriptome shotgun sequencing (RNA‐Seq; Absolute Antibody, Oxford, UK). Contigs were assembled using a proprietary approach and data were mined to identify all viable antibody sequences. Variable heavy (VH) and variable light (VL) domains were identified separately and the relative abundance of each identified variable region gene was reported in transcripts per million. The anticipated species and isotype of the BB5.1 antibody were genetically confirmed. The CDRs for the primary VH and VL sequences were automatically identified working to the Kabat definition for CDRs.

#### Predicting the BB5.1‐targeted epitope on C5 using computational methods

The structure of BB5.1 was predicted using the abodybuilder ^32^ online pipeline (https://omictools.com/abodybuilder‐tool Based on the mouse C5 sequence (https://www.ncbi.nlm.nih.gov/protein/NP_034536.3) and human C5 crystal structure (https://www.ncbi.nlm.nih.gov/protein/AAA51925.1); a coordinate structure of the mouse C5 was predicted using SWISS‐Model.^33^ These two coordinate files were inputted to the EpiPred server and used to predict the most likely region of interaction between BB5.1 and C5.^34^ Using the highest ranked epitope site as the receptor site, these three‐dimensional structures were subjected to docking simulations using the molecular operating environment (MOE; https://www.chemcomp.com/Products.html). The two structures were docked using the protein–protein docking tool and used to identify the most likely epitope on C5 and the key amino acids predicted to be important for binding. The structure with the best score from the docking was selected and this complex was subjected to 100 nanoseconds of molecular dynamic simulation using the GROMACS suite.^35^ The simulation involved placing the structure in a cubic box, solvation using TIP3P‐H_2_O and neutralization using appropriate ions; long‐range electrostatic interactions were modelled using the particle mesh Ewald method and a 1·4‐nm cut‐off was applied to Lennard–Jones interactions. The Amber10 force field (http://ambermd.org/) was selected for simulation. All of the simulations were carried out in the isobaric‐isothermal (NpT) ensemble at 310K with periodic boundary conditions. Each simulation comprised: (i) energy minimization, using the steepest descent method and a tolerance of 1000/KJ/nm; (ii) warm‐up stage of 25 000 steps at 0·002 ‐ps steps with atomic restraint applied to settle the model; (iii) a molecular dynamic stage over a total of 100 nanoseconds. Further to this, a second docking simulation was run using the HADDOCK 2.4 server; all predicted epitope sites were inputted into the server and these were ranked from most to least likely conformer.

## Results

### Cross‐species complement inhibition in haemolytic assays

**Figure 1:**
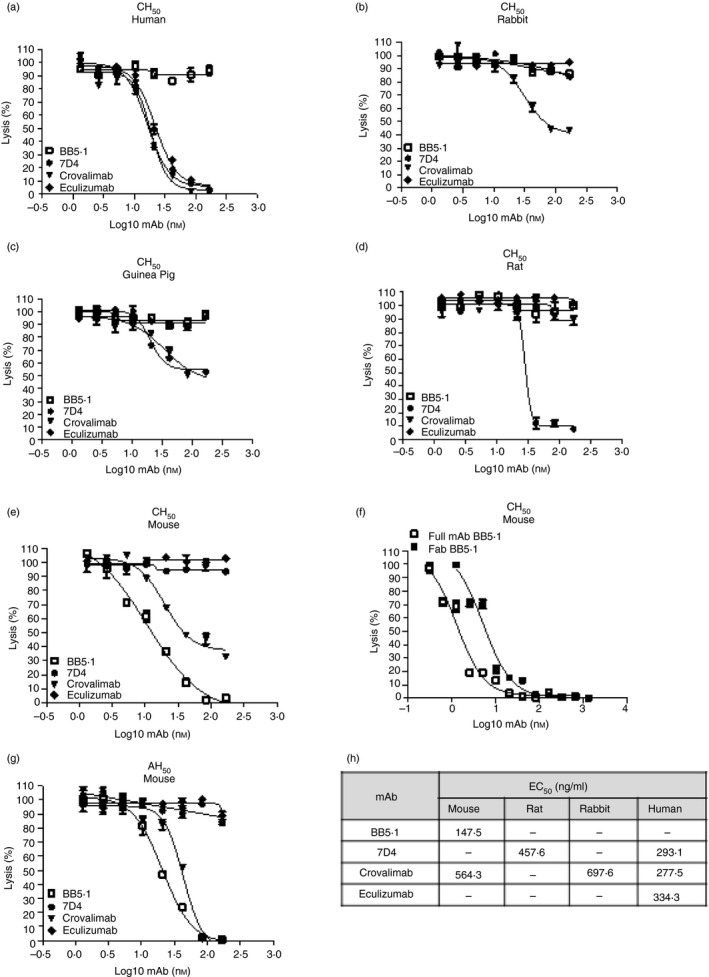
Haemolytic assays to investigate BB5.1 inhibition of complement‐mediated lysis across species. In classical pathway assays (CH50; a–f), sera used were human (a), rabbit (b), guinea pig (c), rat (d) and mouse (e,f). BB5.1 only inhibited haemolysis in mouse serum, confirming that it is specific for mouse C5. Commercial anti‐C5 monoclonal antibody (mAb) crovalimab and eculizumab and in‐house anti‐C5 7D4 were used as comparators. BB5.1‐derived Fab fragment inhibited mouse serum‐mediated haemolysis as effectively as the full‐length BB5.1 (f). BB5.1 also efficiently inhibited mouse serum mediated haemolysis in an alternative pathway assay (AH_50_; g). All tested mAbs except BB5.1 strongly inhibited human C5, 7D4 strongly inhibited rat C5 and partially inhibited guinea pig. Crovalimab weakly inhibited mouse, guinea pig and rabbit complement. The 50% inhibitory doses (EC_50_) for each antibody and serum (except guinea pig as none reached 50% inhibition) were calculated (h). All experiments were repeated three times with comparable results. The error bars are standard errors of triplicates.

**Figure 2:**
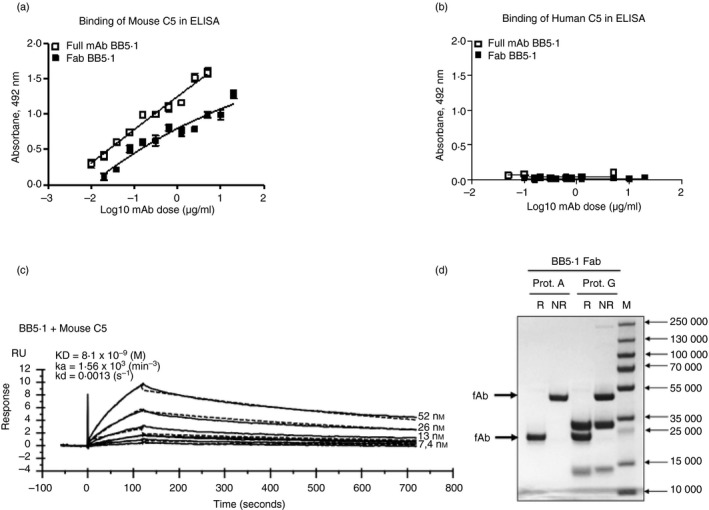
Measuring binding of BB5.1 monoclonal antibody (mAb) to human and mouse C5. In direct ELISA, plates were coated with mouse C5 (a) or human C5 (b); BB5.1 (full‐length IgG and Fab) detected mouse C5 but not human C5 in the assay. Signals obtained for Fab fragments and full mAb were comparable. All experiments were repeated three times with comparable results. The error bars are standard errors of triplicates. (c) BB5.1 was immobilized directly onto a CM5 sensor chip by amine coupling at ~ 200 RU. Mouse C5 was flowed in HBS‐EP at 52–4 nm and interactions with the immobilized mAb were analysed. Sensorgrams were collected and KDs were calculated using the Langmuir 1 : 1 binding model. Representative sensorgrams are shown with fitted data in black (*n* = 3). As expected, BB5.1 bound strongly mouse C5; the association constant (*k*_a_) was 1·56 × 10^5^ min^−1^, the dissociation constant (*k*_d_; *k*_off_) was 0·0013 s^−1^, and the calculated K_D_ was 8·10 × 10^−9^ m. (d) Purity of the Fab was assessed by SDS–PAGE on 4–20% gel (Biorad, Watford, UK; #4561093) under non‐reducing (NR) and reducing (R) conditions, stained with Coomassie blue. As expected, the Fab runs at 50 000 NR and 25000 R. M, molecular weight marker (PageRuler #26620). The contaminant at ~ 30 000 in the protein G lanes represents protein G released from the solid phase during BB5.1 Fab purification; to avoid this issue, protein A was selected for all bulk Fab preparations.

**Figure 3:**
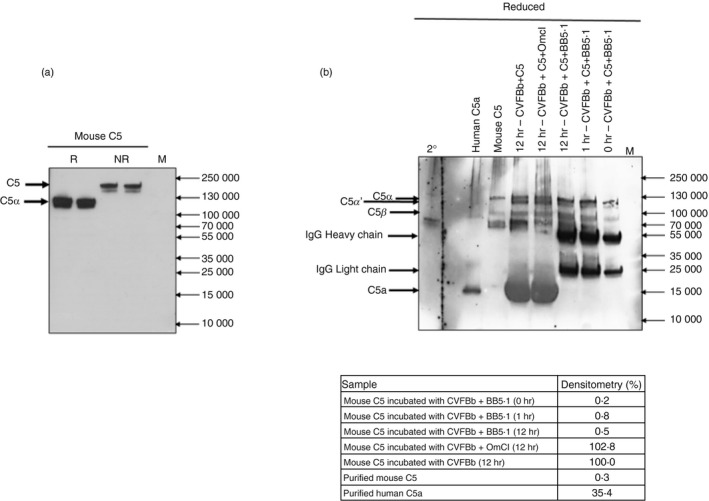
Western blots to confirm BB5.1 C5 binding and test effect on C5 cleavage. (a) Mouse C5 (1 µg) was resolved on 4–20% SDS–PAGE gel (Biorad, #4561093) under non‐reducing (NR) and reducing (R; 5% *β*‐mercaptoethanol) conditions, each in duplicate. The blot was probed with biotinylated BB5.1 (1 µg/ml) and detected with streptavidin‐horseradish peroxidase (R&D Systems, # 890803; 1 : 5000 dilution). Under NR conditions BB5.1 bound intact C5 at 195 000 and a minor band at ~ 180 000, probably a C5 proteolytic fragment. Under NR conditions BB5.1 bound the mouse C5*α*‐chain at 115 000 MW; there was no signal for the C5*β*‐chain (80 000 MW). (b) Cleavage of mouse C5 by CVFBb. Mouse C5, alone or mixed with BB5.1 or OmCI (1 : 5 and 1 : 10 molar excess, respectively) in HEPES‐buffered saline (HBS), was incubated with CVFBb at 10 : 1 (C5 : CVFBb) molar ratio for various periods up to 12 hr. The reaction was stopped by addition of SDS reduced buffer containing 5% *β*‐mercaptoethanol, samples (1 µg) resolved on 4–20% SDS–PAGE gels and Western blotted to detect intact or cleaved C5 and C5a using polyclonal goat anti‐C5 monoclonal antibody (mAb) (CompTech; A220). C5*α* (115 000), C5*α*’ (110 000), C5a (10·4 000). The inset table shows densitometry analysis of the C5a band using imagej (University of Wisconsin‐Maddison, Madison, WI, ), expressed as % relative to C5a generation in the absence of inhibitor (100%); this confirms that BB5.1 efficiently inhibited cleavage of C5; no C5a was detected at any of the incubation times. The C5 inhibitor OmCI did not inhibit C5 cleavage by CVFBb and resultant C5a generation. CVFBb was used at very low amounts in the assays (~ 0·1 µg; 1 : 10 molar ratio with C5). Results are representative of three independent experiments.

BB5.1 (subclass confirmed as IgG1, *κ*‐chain) was tested in CP and AP haemolysis assays using human, rabbit, guinea pig, rat and mouse sera; the mAb efficiently inhibited haemolysis mediated by mouse serum but had no effect in any of the other sera (Fig. 1a–e). Fab fragments generated from BB5.1 (Fig. 2d) also efficiently inhibited mouse serum haemolysis (Fig. 1f). As we have reported before,^29^ eculizumab inhibited only human serum complement, whereas the Roche anti‐C5 mAb crovalimab additionally inhibited mouse, guinea pig and rabbit sera; an in‐house anti‐C5 mAb 7D4^36^ blocked lysis mediated by human, rat and guinea pig sera (Fig. 1a–d). BB5.1 also efficiently inhibited AP haemolysis mediated by mouse serum (Fig. 1g). The weak inhibition of CP and AP haemolysis in mouse serum mediated by crovalimab contrasts with BB5.1 strongly inhibiting both, suggesting that these mAbs have different binding sites; the demonstration (Fig. 3a) that BB5.1 binds the C5*α* chain whereas crovalimab binds C5*β* confirms this.^36^ The calculated 50% complement inhibitory doses (EC_50_) of all mAbs in the different species sera are shown (Fig. 1h).

### Binding of BB5.1 to mouse and human C5

The direct ELISA showed that both intact BB5.1 and Fab fragments bound mouse C5 but not human C5 (Fig. 2a,b). The strong binding of BB5.1 to mouse C5 was confirmed by SPR analysis on immobilized BB5.1 mAb (200 RU) with mouse C5 flowed over; the calculated *K*_D_ was 8·1 × 10^−9^ m (Fig. 2c). The kinetics demonstrated a very slow off‐rate (*k*_d_;* k*_off_; 0·0013 s^−1^) of mouse C5 from mAb BB5.1, explaining why this mAb is such an efficient inhibitor of complement *in vivo*. It was not possible to test Fab fragments in SPR analyses because direct immobilization of Fab on the chip markedly impacted C5 binding activity.

To test whether BB5.1 bound the *α*‐ or *β*‐chain in mouse C5, Western blotting was performed on purified mouse C5; in reduced blots, BB5.1 bound the *α*‐chain in mouse C5 (Fig. 3a).

### BB5.1 inhibits generation of C5a from mouse C5 by the CVF C3/C5 convertase

To test whether BB5.1 inhibited convertase‐mediated cleavage to release C5a, mouse C5 was incubated with the stable C3/C5 convertase CVFBb with or without an excess of BB5.1 for up to 12 hr; C5a generation, detected by Western blot, was used as an index of C5 cleavage. A complete inhibition of CVFBb‐mediated C5a generation was observed when BB5.1 was included, even during a 12‐hr incubation period; in contrast, inclusion of the tick C5 inhibitor OmCI did not reduce C5a generation (equivalent to the positive control; CVFBb+ mouse C5 alone), confirming that it did not block C5 cleavage by the CVFBb convertase, as reported elsewhere.^25^ Densitometric analysis of the C5a band showed >99% inhibition of C5a generation by BB5.1 compared with the positive control (Fig. 3b).

### CDR sequencing of BB5.1 and *in silico* determination of binding epitopes on C5

**Figure 4:**
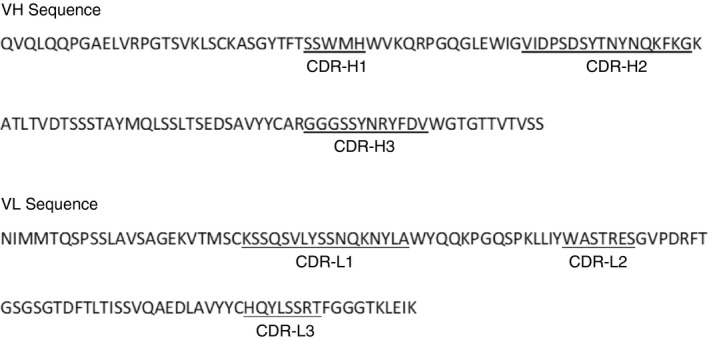
Complementarity determining region (CDR) sequencing of BB5.1. The figure shows the full variable heavy and variable light domain sequences of BB5.1 monoclonal antibody (mAb); the CDRs (underlined and labelled in the variable domain sequences) were identified using the Kabat CDR definitions in an automated program (https://absoluteantibody.com/).

**Figure 5:**
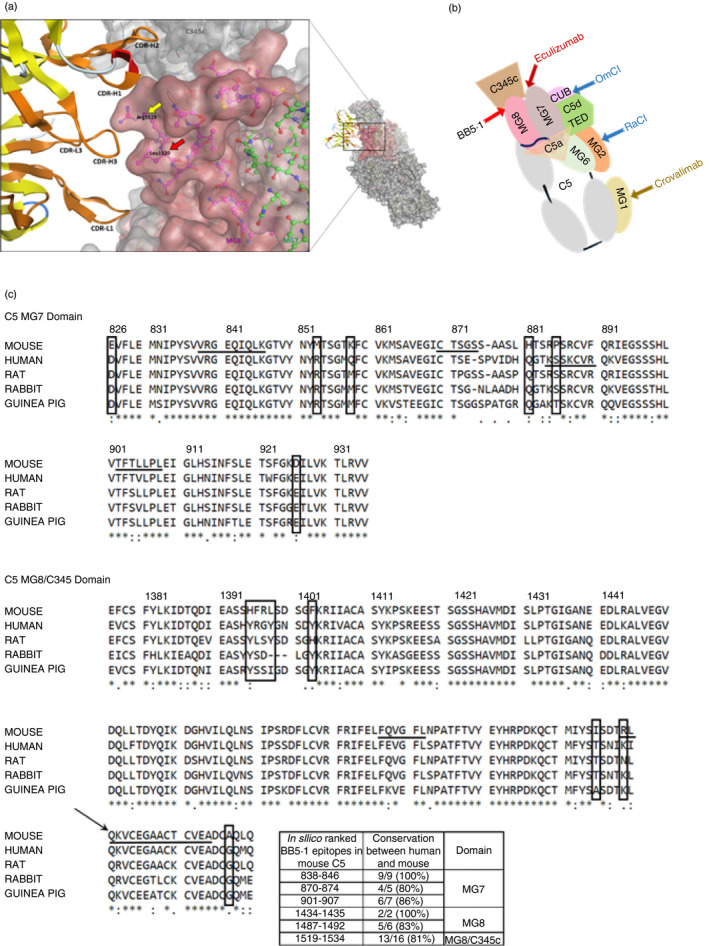
*In silico* modelling of the structure of the C5–BB5.1 complex. (a) Structure of the complementarity determining regions of BB5.1 docked to the predicted best epitope on mouse C5. The model shows that the BB5.1 binding site on C5 involves three domains, MG7, MG8 and C345c domains (in light pink, pink and grey), adjacent in the tertiary structure; the zoomed‐in view identifies the highest ranked interacting amino acids identified *in silico*, R1519 and L1520 (arrowed; respectively yellow and red) located at the interface of the MG8 and C345c domains. In the model, these residues contact BB5.1 CRDs H1 and H3. The interacting CDRs are highlighted in orange. (b) Cartoon diagram showing the domain organization of C5 in the same orientation as the crystal structure in (a), mapping interaction sites of inhibitors; monoclonal antibody (mAb) BB5.1, eculizumab, crovalimab, the tick inhibitors OmCI and RaCI. (c) Alignment of mouse and human, rat, rabbit and guinea pig C5 MG7 and MG8/C345c domain sequences. The arrow marks the interface between MG8 and C345c. Numbering for the mouse sequence is above. An asterisk indicates positions that have a single, fully conserved residue. A: (A followed by a colon) indicates conservation between groups of strongly similar properties – scoring >0·5 in the Gonnet PAM 250 matrix. A. (A followed by a period) indicates conservation between groups of weakly similar properties– scoring ≤0·5 in the Gonnet PAM 250 matrix. *In silico* predicted BB5.1 epitopes are underlined in the mouse sequence; the eculizumab epitope (K879–R885) is underlined in the human sequence. The mouse‐specific residues are highlighted in boxes. The inset table lists the *in silico* ranked BB5.1 epitopes in mouse C5 MG7, MG8 and C345c domains, with percentage conservation between human and mouse. Alignments were performed using Uniprot clustal o(1.2.4) software (EMBL‐EBI, Cambridge, UK; https://www.uniprot.org/align/) with the following sequences; mouse: P06684, human: P01031, rat: A0A1B0GWS5, rabbit: G1SPF9, guinea pig: H0UY41.

The derived sequences of the BB5.1 CDRs are shown in Fig. 4. The *in silico* modelling confirmed binding of BB5.1 to mouse C5*α* chain and predicted the likely binding sites (Fig. 5a,b). The modelling predicted several sequences in the MG7, MG8 and C345c domains as potential epitopes (see insert table in Fig. 5c). Several of these predicted epitopes, including a predicted epitope site at 870–874 (Fig. 5c), were 100% conserved between mouse C5 and C5 from other species and so are unlikely to be key binding epitopes for this mouse C5‐specific mAb. As expected, mouse and rat C5 sequences were highly homologous with the following residues in the regions of interest being specific for mouse only; 826E, 853M, 858K, 881H, 885P, 926D, 1395H, 1396F, 1397R, 1398L, 1403F, 1515I, 1519R, 1537A. We therefore propose that the most likely epitope site is that surrounding 1519R, where docking simulations from both MOE and HADDOCK predicted that R1519 and L1520, located in the MG8 domain at the interface with the C345c domain, are key interacting residues, closely associated with CDRs H1 and H3 in BB5.1 (Fig. 5a). These domains represent a critical region in C5 activation and MAC formation, involved in C5 interaction with the C5 convertase and also contributing to the interacting surface between C5b and C7 during MAC assembly.^37^, ^38^

## Discussion

Although BB5.1 was the first complement activation blocking mAb developed, and has been used in many laboratories over many years,^9^ little was known about its mechanism of C5 inhibition. Here, we have characterized BB5.1 and demonstrated that its function‐blocking activity is mouse specific, that it inhibits in both CP‐mediated and AP‐mediated lysis assays, and that inhibitory activity is retained in BB5.1‐derived Fab fragments (Fig. 1f–g). The strong binding of BB5.1 to mouse C5 was confirmed by ELISA, Western blotting and SPR analysis, the latter demonstrating the strength and tenacity of binding (*K*_D_ = 8·10 × 10^−9^ m with slow off‐rate; *k*_off_ 0·0013 s^−1^ Figs 2a,c and 3a). BB5.1 showed no interaction with human C5 in ELISA (Fig. 2b) or SPR (negative data not shown), confirming the lack of any binding activity.

The capacity of Fab fragments derived from BB5.1 to efficiently inhibit C5 function establishes that intact antibody is not necessary for function and smaller, active fragments may prove useful as tools for therapy studies. Fab fragments are approximately 50 000 in mass, about a third the size of intact antibody, and this may confer advantages in accessing some tissue sites – for example, the brain. Sequencing of the CDRs of BB5.1, reported here, enables the generation of even smaller recombinant fragments; recombinant single‐chain variable fragments have a mass of ~ 27 000 and are reported to cross the blood–brain barrier.^39^, ^40^ Generating single‐chain variable or other small recombinant fragments from the CDRs of BB5.1 may allow proof‐of‐concept studies for C5 blockade in diseases of the central nervous system where the blood–brain barrier is grossly intact, blocking ingress of intact mAbs.

BB5.1 blocked the cleavage of mouse C5 by the stable C3/C5 convertase CVFBb and consequent C5a generation; the tick‐derived C5 inhibitor OmCI, which binds epitopes in the C5d, CUB and C345c domains, did not inhibit the CVFBb convertase as previously reported (Fig. 3b).^25^ These data, together with published work,^25^, ^38^ further support the concept that C5 has multiple surface epitopes that can be targeted by mAb or other agents to cause inhibition of C5 cleavage and/or function, probably through steric effects such as conformational locking.^25^

*In silico* modelling of the interaction of BB5.1 with mouse C5 predicted specific interactions with residues in the MG7, MG8 and C345c domains of the C5*α*‐chain, co‐localized in a binding face on the molecule (Fig. 5). Eculizumab also binds the C5*α*‐chain in a complex manner with its key epitope located to K879 to R885 in MG7, but with other contributing residues from outside MG7, including the *β*‐hairpin residues S913 to I922 that include T916, W917, which are unique to human C5 and may explain the human specificity of eculizumab.^26^, ^27^ Individuals expressing C5 with the R885H polymorphism, common in Japan with a prevalence of 3·5%, are resistant to C5 blockade using this drug.^41^ The proximity of binding sites for BB5.1 (predicted site) and eculizumab suggests that they may have a similar mode of action; however, further analyses, including mutations of predicted key binding residues, are needed to confirm this possibility.^4^, ^25^, ^38^ Eculizumab is highly specific for human C5 whereas BB5.1 is specific for mouse C5 with no binding or activity toward C5 from other species, including other rodents (Fig. 1). To better understand these species restrictions, the sequences of mouse, human, rat, rabbit and guinea pig C5 across the key binding domains for the two mAb were aligned (Fig. 5c). There was limited conservation between human and mouse C5 in the key eculizumab epitope, including a shape‐shifting proline substitution unique to mouse (KSSKCVR versus RPSRCVF) and some of the predicted BB5.1‐interacting residues in mouse C5 were conserved in both human and rat C5. Importantly, the key predicted interacting residues in mouse C5, R1519 and L1520, were replaced by K and I in the human sequence.

In summary, we show that BB5.1 and eculizumab bind the C5*α*‐chain at similar sites, the former predicted to involve domains MG7, MG8 and C345c, the latter with a primary epitope in MG7, and inhibit complement in the same manner, blocking C5a generation as well as MAC formation and acting as conformation locks, preventing the structural ‘priming’ event that is necessary for the cleavage. The work provides a mechanistic basis for the activities of this landmark antibody, a much‐used tool that was a major impetus to the development of anti‐complement drugs for human disease.

## Author contributions

WMZ performed all the laboratory analyses and wrote the first draft of the manuscript. GM performed the *in silico* modelling and docking analysis. AB provided critical analysis on the computing modelling. BS generated and donated the BB5.1 cell line. BPM conceived and planned the study and oversaw the data handling and manuscript preparation. All authors contributed to and have approved the final manuscript.

## Disclosure

BPM has provided advice on complement to Roche and is a consultant to RaPharma. Other authors declared no potential conflicts of interest with respect to the research, authorship and/or publication of this article.
